# Post‐Embryonic Development and Formation of the Heterocoelic Aquiferous System in Two Species of Calcareous Sponges (Calcarea, Porifera)

**DOI:** 10.1002/mrd.70060

**Published:** 2025-10-03

**Authors:** Emilio Lanna, Michelle Klautau

**Affiliations:** ^1^ Instituto de Biologia Universidade Federal da Bahia Salvador Bahia Brazil; ^2^ National Institute of Science and Technology in Interdisciplinary and Transdisciplinary Studies in Ecology and Evolution (INCT IN‐TREE) Salvador Bahia Brazil; ^3^ Zoology Department, TaxoN Laboratory, Avenida Carlos Chagas Filho Federal University of Rio de Janeiro, Biology Institute Rio de Janeiro Rio de Janeiro Brazil

**Keywords:** choanocytes, epithelium, evolution, metamorphosis, morphogenesis

## Abstract

We characterized the morphogenetic processes of larval metamorphosis and the development of the olynthus and heterocoelic aquiferous system (AS) in *Sycettusa hastifera* (syconoid) and *Paraleucilla magna* (leuconoid) (Porifera, Calcarea). Metamorphosis and development up to the olynthus were similar in both species. During metamorphosis, apparently, the juvenile's cell lineages were established by invagination of the ciliated pole into the larval cavity, while posterior pole cells covered the juvenile. Ciliated cells seemed to differentiate into an inner cell mass (ICM) (forming choanoblasts and scleroblasts), while granular cells seemed to form mainly the pinacoderm. Then, cavitation of the ICM formed the asconoid AS of the olynthus. Choanocyte chamber morphogenesis for the syconoid and leuconoid AS seemed to be epithelial, but followed different paths. In *S. hastifera*, the chambers were formed by folding the primordial choanoderm towards the external portion of the sponge, whereas in *P. magna*, invagination of the choanoderm into the spongocoel segregated groups of choanocytes into spherical chambers. The morphogenesis of the heterocoelic AS in these calcareans is likely different from that in Demospongiae (leuconoid AS is established via mesenchymal‐to‐epithelial morphogenesis) but similar to Homoscleromorpha (AS arises through epithelial morphogenesis). Characterizing the post‐embryonic development in these species is the first step toward understanding the mechanisms that regulate the ontogeny and evolution of Porifera's primary synapomorphy: the aquiferous system.

## 1 Introduction

Animal development does not cease after the cell divisions and movements (e.g., morphogenesis and gastrulation) that generate embryos (Gilbert and Barresi 2016). Nearly all metazoan phyla exhibit an intermediate life stage (larva) that differs in form and behavior from the adult. This difference is particularly pronounced in benthic marine invertebrates, where the larval stage is free‐swimming in the water column (plankton), while adults reside on the seabed (e.g., Hall and Wake 1999). To transition from the free‐swimming to the benthic phase, larvae undergo radical changes in tissue and organ organization, a process known as metamorphosis (Hall and Wake 1999; Gilbert and Barresi 2016). Due to the evolutionary position of Porifera among metazoans, it has been suggested that understanding the processes and mechanisms of sponge metamorphosis could shed light on how morphogenesis and the establishment of cell lineages arose during the evolution of multicellular animals (Gonobobleva and Ereskovsky 2004; Leininger et al. 2014; Nakanishi et al. 2014).

Simpson (1984) highlighted that despite the aquiferous system being responsible for all sponge primary physiology, surprisingly few studies have investigated its structure, composition, and function across all Porifera groups. This statement is still true even after 40 years, especially if its development is considered. The development of the aquiferous system has been overlooked by sponge researchers, with works mainly describing the formation of the initial aquiferous system (reviewed in Ereskovsky 2010). In demosponges and homoscleromorphs, their typical aquiferous system (in homoscleromorphs some variation may be observed, but most species are leuconoid, as in demosponges) develops as a single step in the end of the metamorphosis (Ereskovsky et al. 2007; Nakanishi et al. 2014; Leys et al. 2025). The class Calcarea, on the other hand, is particularly well‐suited for investigations aiming to describe the development of different types of aquiferous systems, as it presents the largest diversity of this important trait of sponges. Currently, six types of aquiferous systems are observed in the adults of Calcarea: asconoid, syconoid, sylleibid, leuconoid, solenoid, and kladonoid (Cavalcanti and Klautau 2011; Lopes and Klautau 2023). The varying degrees of complexity in Calcarea led sponge biologists to propose different evolutionary pathways based on the organization of the aquiferous system, suggesting an evolution from simpler to more complex forms (Haeckel 1872; Borojević et al. 2002). However, this idea was not accepted by all the authors (Bidder 1898) and phylogenies generated with DNA sequences showed that indeed the aquiferous system does not follow an evolutionary path from simple to complex forms (Manuel et al. 2003; Manuel 2006; Dohrmann et al. 2006; Voigt et al. 2012). For example, Voigt et al. (2012) proposed that morphological simplifications, including the simplification of the skeleton and convergent evolution of aquiferous systems, occurred during Calcarea evolution.

From an ontogenetic perspective, all calcareous sponges, regardless of their aquiferous system type in adulthood, pass through an initial stage (olynthus) where the aquiferous system corresponds to the asconoid (Haeckel 1872; Minchin 1900; Tuzet 1973; Amano and Hori 1993; Ereskovsky 2010). This is true independently of whether the development derives from a larva or from cell aggregates (primmorphs) passing through somatic embryogenesis (Lanna and Klautau 2019). Despite being crucial for understanding the evolution of this animal group, the formation of the aquiferous system is still poorly known. Only Maas (1900), for example, investigated the formation of the syconoid aquiferous system, and the formation of systems such as sylleibid, solenoid, kladonoid, and leuconoid remains entirely unknown.

In this study, we investigate how the amphiblastula larva transforms into an olynthus and how it develops in a syconoid (*Sycettusa hastifera* Row, 1909) or leuconoid (*Paraleucilla magna* Klautau, Monteiro and Borojević, 2004) sponge. Our objective was to characterize the morphogenetic processes occurring during metamorphosis, post‐embryonic development, and the acquisition of heterocoely in these species, laying the foundation for future studies that will aid in understanding the molecular and cellular mechanisms governing this stage of sponge ontogeny. Additionally, this characterization will enable us to seek potential ontogenetic explanations for the differentiation and evolution of aquiferous systems, the main synapomorphy of Porifera.

## 2 Materials and Methods

### 2.1 Investigated Species

Both species investigated in this study belong to the subclass Calcaronea (Calcarea, Porifera) and were abundant in the environments where they were collected. *Sycettusa hastifera* is a syconoid sponge that has a cortex covering its radial choanocyte chambers. This species was easily found at Forno Beach, Arraial do Cabo, Rio de Janeiro, Brazil, where it is considered invasive. This sponge reproduces semi‐continuously, with peaks of larval release spaced throughout the year. It is also able to reproduce asexually from cell aggregates in vitro (Lanna and Klautau 2018, 2019, 2022). The other species investigated here, *P. magna*, is also considered invasive in Brazil. This species has a leuconoid aquiferous system with spherical chambers scattered within a thick choanosome (see Lanna and Klautau 2010, 2012). *Paraleucilla magna* reproduces continuously, with constant larval release throughout the year (Lanna et al. 2015).

### 2.2 Collection of Adults and Maintenance of Juveniles

Adult specimens of both species were collected between March 2007 and July 2011 (Table 1). Individuals were carefully removed from the substrate using a knife, minimizing damage to their integrity. Immediately after removal, while still underwater, the specimens were placed in plastic containers with seawater and transported to the laboratory. The individuals were transferred to beakers with approximately 400 mL of seawater and monitored under a stereomicroscope until larval release began. The beakers were maintained at room temperature (ca. 25°C) without aeration, as these conditions stimulate larval release. Larvae were collected from the water surface directly using sterile Petri dishes or Pasteur pipettes. On some occasions, larvae were centrifuged (< 1000 rpm, 5 min) in sterile test tubes to increase their concentration.

**Table 1:** Summary of data on the collections of individuals, abundance of larvae of *Sycettusa hastifera* and *Paraleucilla magna*, and analysis method for the characterization of post‐embryonic development in this study.

Date	Collected species^a^	Location	Qualitative assessment of the abundance of released larvae^b^	Analysis method^c^
Mar/07	PM	Rio de Janeiro	High	IM
Apr/07 (2 collections)	PM	Rio de Janeiro	High	TLVD, Fix
Mar/08	PM	Rio de Janeiro	High	TLVD, Fix
May/09	PM	Rio de Janeiro	Low	TLVC
Aug/09	SH, PM	Arraial do Cabo	Low	IM, TLVC
Sep/09 (2 collections)	SH, PM	Arraial do Cabo and Rio de Janeiro	High	IM, TLVC
Sep/10	SH, PM	Arraial do Cabo	Low	IM, Fix
Nov/10	SH, PM	Arraial do Cabo	Low/High*	IM, Fix
Jan/11	SH	Arraial do Cabo	Absent	—
Feb/11	SH	Arraial do Cabo	Absent	—
Mar/11	SH, PM	Arraial do Cabo	Absent/Moderate*	IM, Fix
Jul/11	SH, PM	Arraial do Cabo	Absent/Low*	IM, Fix

^a^ SH, *Sycettusa hastifera*; PM, *Paraleucilla magna*.

^b^ Due to the large number of larvae released by the species, quantification of these reproductive elements was not feasible. Thus, we opted for a qualitative assessment of the abundance of released larvae that could vary from: Absent (no larvae released); Low (< 200 larvae); Moderate (200 < larvae < 1000) or High (> 1000 larvae). (*) The first abundance refers to *S. hastifera*, while the second refers to *P. magna*.

^c^ IM, inverted microscope with phase contrast; Fix, material fixed in Bouin; TLVC, time‐lapse videos microscopy with images obtained at time intervals using phase contrast microscopy; TLVD, time‐lapse videos with images obtained at time intervals using differential interference contrast microscopy (DIC).

Subsequently, the larvae were placed in sterile 60 mm diameter plastic Petri dishes. Glass coverslips (24 × 24 × 0.17 mm) were placed at the bottom of some dishes to facilitate specimen fixation and staining for microscopic observation (see below). For juvenile maintenance, the water in the dishes was changed every 2 days, and aeration was provided daily using an aquarium pump attached to a Pasteur pipette with its tip inserted into the water. After 1 week, the dishes were transferred to a 60 L aquarium with circulating seawater, from the locality where they were collected, operating in a closed system. The placement of the dishes in the aquarium was intended to increase nutrient availability for the sponges.

### 2.3 Characterization of Post‐Embryonic Development

The behavior of the amphiblastula larvae, the metamorphosis, and formation of the aquiferous system were observed in vivo using a range of microscopy techniques. The release and swimming activity of the amphiblastulae were monitored using a stereomicroscope. To characterize metamorphosis and aquiferous system development, we used inverted microscopes equipped with phase contrast (PC) and differential interference contrast (DIC), which provided better in vivo cell visualization. Images were captured using digital cameras attached to the microscopes. Time‐lapse videos and their subsequent analyses were performed using the tool “Stack animation” in the ImageJ software (http://rsb.info.nih.gov/ij/index.html). For a finer description of the different stages of development, the coverslips where the juveniles attached and started their development were fixed at different time intervals using Bouin's fixative solution for 6 h at room temperature (Lanna and Klautau 2022). The use of this fixative removed the very thin spicules (when present) of the juveniles. The fixed specimens were stained with acid carmine (CA) or Harris's hematoxylin and eosin (HE) and observed and photographed whole mount (*in toto*) under a compound microscope. Virtual sections and 3D reconstructions were obtained in a confocal laser scanning microscope (Zeiss LSM510 Meta) (LSCM) using He 488 nm Argon/2 laser and LP 505 filter, following the methodology applied by Lanna and Klautau (2012). Some specimens were also observed in the Transmission electron microscope. For this, the samples were fixed in a solution containing glutaraldehyde 25%, 0.2 mol.L^−1^ sodium cacodylate buffer, and filtered seawater (1:4:5 proportion, respectively – final concentration of glutaraldehyde: 2.5%) at 4°C. Samples were post‐fixed with 1% osmium tetroxide and 1% potassium ferrocyanide for 30 min at room temperature, washed in the sodium cacodylate buffer, dehydrated in an acetone series, and embedded in Epon resin. Then, we carried out ultrathin sections (ca. 90 nm), contrasted with 5% uranyl acetate for 40 min and lead citrate for 5 min. Finally, the preparations were observed on a ZEISS EM900 EX Transmission Electron Microscope (Lanna and Klautau 2012).

## 3 Results

Under normal conditions, *P. magna* released more than 1000 larvae per sponge within a 1–3‐h period. Apparently, there was no preference for the time of day or lunar phase for larval release in this species, as the spawning usually occurred about 3 h after the sponge was collected. In contrast, the release of larvae from *S. hastifera*, while also occurring in large numbers (over 1000 larvae per sponge), was rarer and apparently happened mostly in the morning after collection, regardless of the collection time (Table 1).

**Figure 1:**
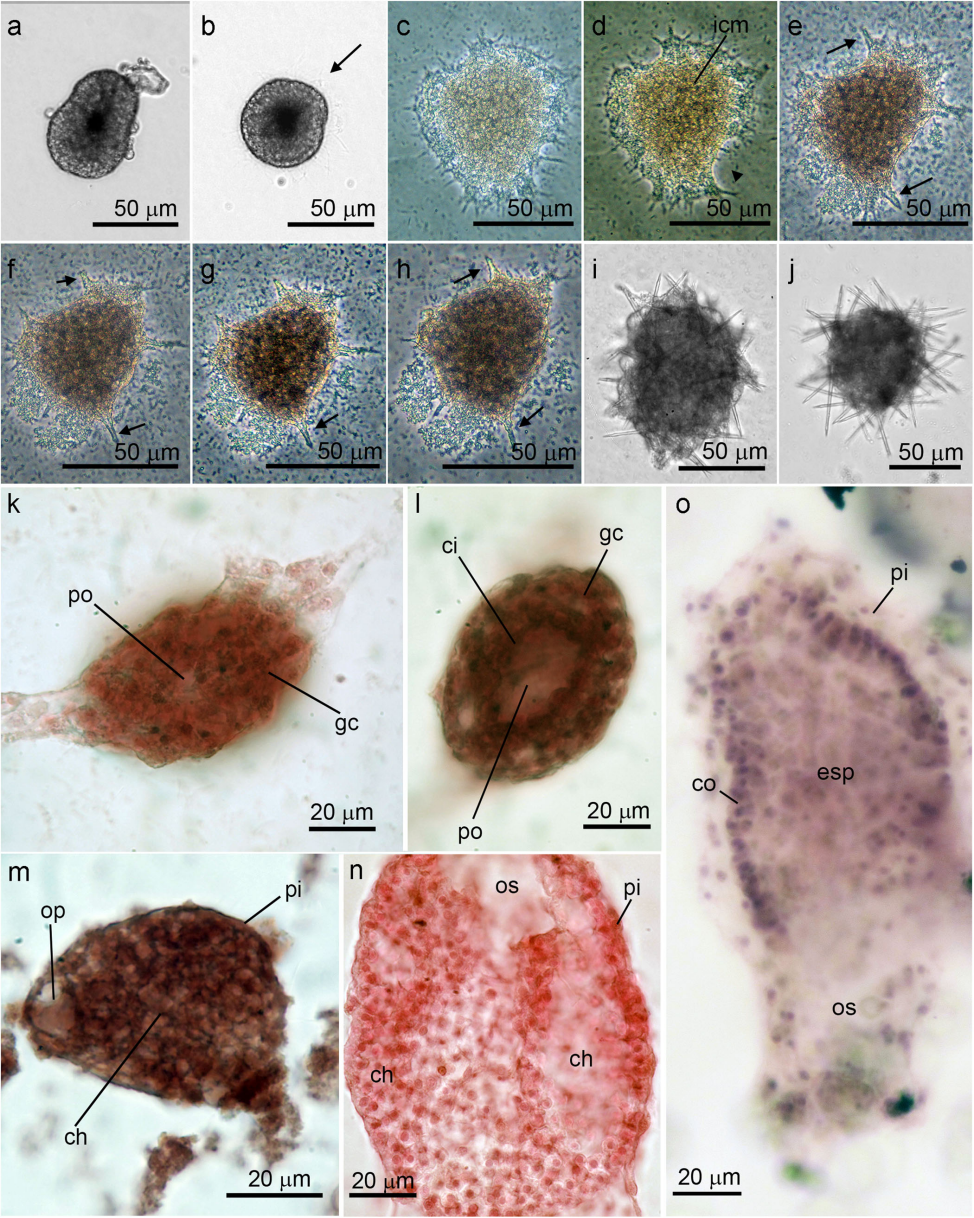
Early post‐larval stages of *Sycettusa hastifera* and the formation of the syconoid aquiferous system. (a) Amphiblastula on the water surface (Phase contrast: PC). (b) View of the posterior pole of the larva at the moment when the anterior pole was testing the substrate with cilia (arrow) (PC). (c–h) Time‐lapse photographs showing the process of the juvenile's spreading shortly after metamorphosis (PC): (c) T = 0, post‐metamorphosis; (d) T = 30 min (retraction of the spreading region – arrowhead – and the formation of inner cell mass (icm)). (e) T = 60 min ‐ first spicules (arrow) being secreted; (f) T = 120 min; (g) T = 6 h; (h) T = 12 h. (i) T = approximately 12 h post‐settlement, still in the spreading form, showing many diactines emerging from the body (PC). (j) Hispid olynthus 24 h post‐settlement (PC). (k–l) Two focal planes of the same individual fixed during the metamorphosis. The granular cells (gc), located around the pore (po) through which the ciliated cells (ci) entered and spread to attach the individual to the substrate (carmine ‐ CA). (m) Juvenile 24 h post‐settlement, flattened and covered by pinacocytes (pi), with the choanoderm (ch) opening into a primordial osculum (op) (CA). (n) Side view of a juvenile 5 days post‐settlement, showing signs of division of the primordial aquiferous system, with choanocyte chambers (cc) being formed. The individual already had a relatively large osculum (os) (pi – pinacocytes, CA). (o) Histology of the olynthus with a single‐layered choanoderm (ch) covered by pi (hematoxylin‐eosin, HE).

**Figure 2:**
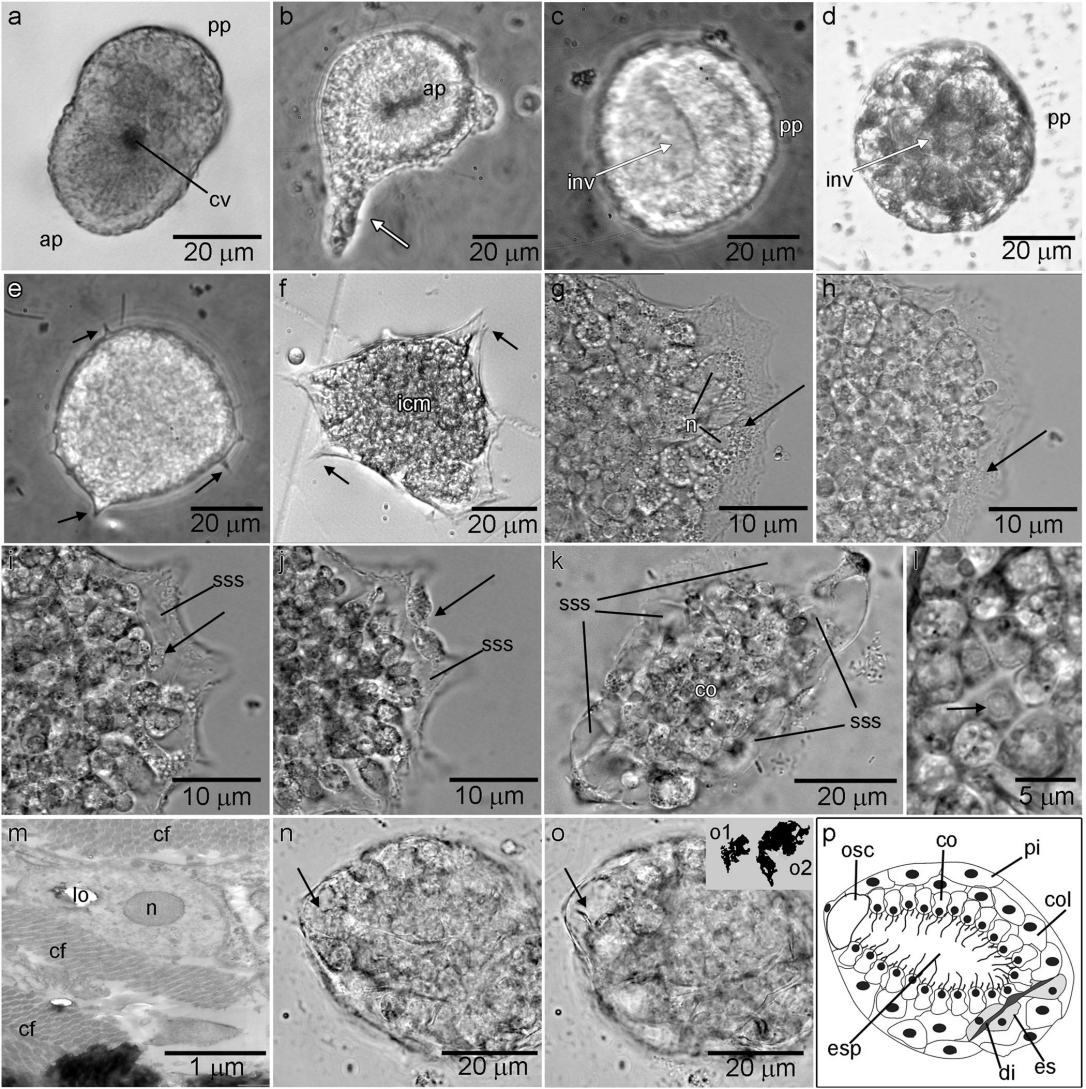
Metamorphosis and early post‐larval stages of *Paraleucilla magna*. (a) Amphiblastula with the anterior pole (ap) composed of ciliated cells and the posterior pole (pp) consisting of granular cells (cv – internal cavity of the larva) (Phase contrast: PC). (b) Deformed amphiblastula (arrow) after centrifugation (PC) (ap – anterior pole) (PC). (c) Beginning of the invagination (inv) of the ciliated cells into the larval cavity (pp – posterior pole, PC). (d) Post‐larva with a temporary pore (inv) formed during invagination (PC). (e) Granular cells projecting filopodia (arrows) for substrate adhesion after pore closure (PC). (f) Spreading of the juvenile with the emission of long pseudopodia (arrows) and differentiation of the inner cell mass (ICM) (differential interference contrast: DIC). (g) At the beginning of the spreading, the cells of the recently settled juvenile show a large number of granules in their cytoplasm (arrow) (n – nucleus, DIC). (h) 1 h post‐settlement, cells derived from granular cells (arrow) secreting extracellular matrix to form the subsurface space (DIC). (i) 2 h post‐settlement, the subsurface space (sss) (future mesohyl) was already formed. Lophocytes were moving rapidly in this region (arrow) (DIC). (j) Cells from the inner cell mass divide (arrow) and differentiate into basopinacocytes (2 h post‐settlement) (DIC). (k) Approximately 6 h post‐settlement, choanocytes began to differentiate in the inner cell mass and formed the choanoderm (co). Many subsurface spaces (sss) were found at this stage (DIC). (l) Detail of the choanoderm showing the apical region of choanocytes with the collar (arrow) surrounding the flagellum (DIC). (m) TEM of a lophocyte (lo) in the subsurface space secreting collagen fibrils (cf) (n – nucleus). (n–o) Same juvenile photographed in an interval of 2 h showing the formation of the primordial osculum of the juvenile. In ‘n’, pinacocytes are moving apart to form the osculum (arrow), which increases 2x in approximately 2 h, as shown in ‘o’. The insert in (o) shows the shades corresponding to the area of the osculum in the figure ‘n’ (*o1*) and in figure ‘o’ (*o2*), showing the increase in this region of the juvenile (DIC). (p) Diagram of a cross‐section of the juvenile approximately 12 h post‐settlement with an asconoid aquiferous system (cc – choanocyte chamber, co – choanocytes, col – colencyte, di – diactine, es – sclerocyte, osc – osculum, pi – pinacocyte).

In both species, the amphiblastula larvae were released through the osculum. The larval morphology of both species was similar, consisting of an epithelium formed by ciliated cells, at the anterior pole, cross‐cells at the equator, and granular cells, at the posterior pole. However, the cilia of the amphiblastula from *S. hastifera* appeared to be longer, and these larvae were slightly larger (ca. 50 μm in *S. hastifera* and ca. 35 μm in *P. magna* [Figures 1a and 2a]). After being released, the larvae of both species swam to the water surface, where they rotated around their own axis in a clockwise direction and moved in large circles at the surface (Supporting Information: Video S1). The swimming duration ranged from just over an hour to more than 3 days, but typically lasted about 3 h for *P. magna* and 12 h for *S. hastifera*. Larvae concentration via centrifugation did not appear to affect the free larval behavior, metamorphosis, or juvenile development in either species, although it occasionally resulted in changes in the shape of the larva (Figure 2b).

After this period in the water column, larvae of both species sank and moved along the substrate using the cilia located at the anterior pole. During this benthic period, the amphiblastulae tested the substrate by touching it with their anterior (ciliated) region, while rotating around their own axis. Some larvae were more selective, while others began metamorphosis after testing the substrate only once.

### 3.1 Post‐Embryonic Development of *S. hastifera*

Larvae of *S. hastifera* underwent metamorphosis after a prolonged period testing the substrate (Figure 1b). During metamorphosis, the anterior (ciliated) region of the larva invaginated into the larval cavity. This invagination closely resembles what was observed and described for *P. magna* (see below). The completion of metamorphosis took less than 10 min, marking the first transition phase between the larval stage and juvenile development. At this stage, the granular cells covering the newly settled juvenile spread out, expanding lobopodia peripherally as they differentiated into pinacocytes and adhered the juvenile to the substrate (Figure 1c,k,l). Simultaneously, the cells that had entered the larval cavity apparently left the epithelial organization and formed an inner cell mass (Figure 1d). About half an hour after the spreading stage began, the juvenile started retracting its peripheral extensions (Figure 1d). The first diactine spicules were produced in the inner cell mass and began protruding from the body approximately 1 h after settlement (Figure 1e), when the spreading region retracted completely, and the juvenile acquired a spherical shape (Figure 1f).

Within the next 2 h, the juveniles became hispid, with many diactines projecting from the juvenile's body (Figure 1g–i). By the end of the first day post‐settlement, individuals had developed a primordial aquiferous system. This system was formed through a cavitation process of the inner cell mass and consisted of a single choanocyte chamber that opened into a primordial osculum (Figure 1m). The olynthus was characterized by its elongated cylindrical shape and the appearance of a highly hispid apical osculum (Figure 1j). The olynthus became hispid with many fusiform (both tips were pointed) diactines. By the end of the second day post‐settlement, the first triactine spicules appeared, resembling the cortical triactines found in adults. The olynthus had a large spongocoel lined by active choanocytes (Figure 1o). After approximately 5 days, the choanoderm folded towards the external region of the sponge, forming what appeared to be the first radial choanocyte chambers (Figure 1n).

### 3.2 Post‐Embryonic Development of *P. magna*

The metamorphosis of *P. magna* occurred rapidly (approximately 10 min) while the larva adhered to the substrate. This metamorphosis was characterized by the invagination of the ciliated region of the larva, creating a pore at the site of the invagination (Figure 2c,d and Supporting Information: Video S2). This pore was soon closed by the movement of the granular cells that would form the basopinacoderm of the juvenile (Figure 2e). Based on the in vivo observations and the time‐lapse videos, we suggest that the granular cells differentiated into the exopinacoderm of the juvenile, while the ciliated cells, after entering the larval cavity, formed an inner cell mass (ICM), in which the cells moved isolated from each other (see below).

**Figure 3:**
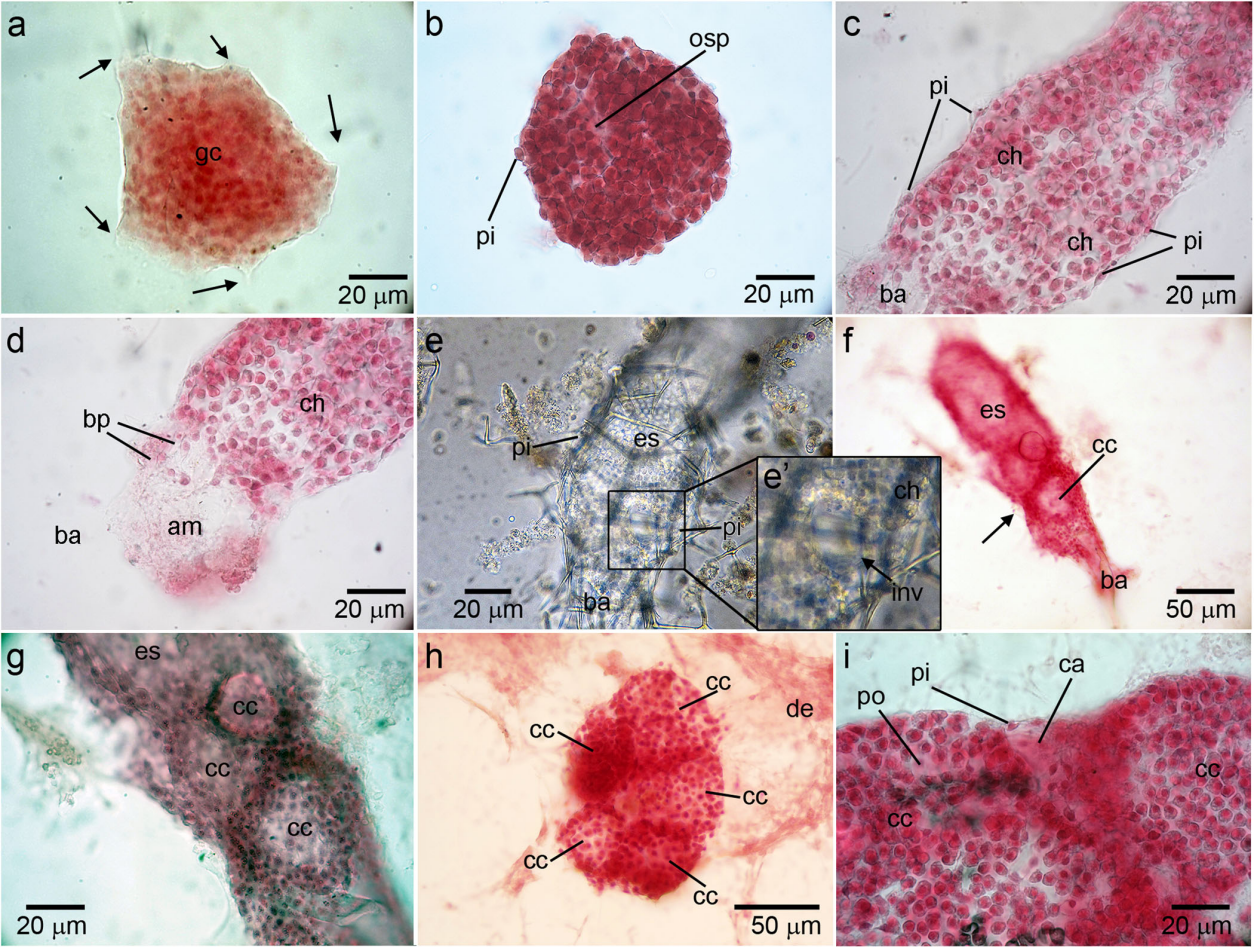
Development of the leuconoid aquiferous system of *Paraleucilla magna*. (a) 2 h post‐settlement, the larva flattened with granule cells (gc) adhering to the substrate (arrow) (Phase contrast: PC). (b) Sponge 48 h post‐settlement, with a primordial osculum (op) and covered by pinacocytes (pi) (PC). (c, d) Side view of a sponge 5 days post‐settlement (hematoxylin and eosin: HE). (c) Apical portion of this olynthus internally coated with choanocytes (ch) and externally by pinacocytes (pi) (ba – base). (d) Detail of the basal portion of the sponge with some basopinacocytes (bp) and the amorphous material (am) attaching the juvenile to the substrate (ch – choanocytes). (e, e') Beginning of the formation of choanocyte chambers through the invagination (inv) of the choanoderm into the spongocoel (es) (ba – base, pi – pinacoderm) (PC). (f, g) Juvenile 17 days post‐settlement (HE): (f) The invagination of the choanoderm reached the opposite side of the choanoderm (arrow), isolating the choanocytes in a choanocyte chamber (cc) (ba – base, es – spongocoel). (g) Detail of the region with choanocyte chambers (cc) indicating the relationship of the choanocyte chambers at the onset of their formation (es – spongocoel). (h–i) Juvenile 20 days post‐settlement (HE). (h) Several choanocyte chambers (cc) formed by successive invaginations of the choanoderm (de – cellular debris deposited in the coverslip). (i) Detail of the choanocyte chambers (cc) that were beginning to separate, forming inhalant canals (ca) (pi – pinacocytes; po – apopyle).

Shortly after metamorphosis, the juveniles spread out on the substrate for their settlement. The marginal cells of the juvenile extended pseudopodia in all directions, flattening the newly settled individual (Figures 2f and 3a). During this spreading, the cells (both from the ICM and the pinacoderm) contained a significant number of reserve granules (Figure 2g), and the inner cells displayed intense movement. Some cells derived from the granular cells of the larva secreted a large amount of extracellular matrix in the peripheral region of the juvenile (Figure 2h and Supporting Information: Video S3). Approximately 3–4 h after settlement, the number of reserve granules and cellular movement decreased. Subsurface spaces began to be formed around 5 h after settlement. In this subsurface space, small cells (about 2 μm), probably derived from the ICM, moved rapidly (Figure 2i,m and Supporting Information: Video S3). They likely also secreted the extracellular matrix, transforming this space into mesohyl, and could be considered as lophocytes (Figure 2m). At the same time, some pinacocytes divided at the peripheral region of the sponge, allowing the juvenile to grow (Figure 2j and Supporting Information: Video S4).

About 6 h after settlement, the juvenile displayed the ICM (where the first choanocytes were differentiating [Figure 2l]) completely surrounded by the mesohyl (Figure 2k). Choanocytes became active, beating their flagella steadily, but at first without a regular orientation (Supporting Information: Video S5). Meanwhile, some openings (probably ostia) on the juvenile's surface (not shown) began to appear. The osculum began to form approximately 7 h after settlement with the movement of some pinacocytes, creating an opening with ca. 10 μm in diameter (Figures 2n and 3b). 1 h later, this opening doubled in diameter and began to constitute the future apical region of the juvenile. With the juvenile's development, this opening functioned as the primordial osculum. This osculum was in contact with the choanoderm in a “pouch‐like” formation differentiated within the ICM through a cavitation process, forming the primordial asconoid aquiferous system (Figure 2o and Supporting Information: Video S6).

**Figure 4:**
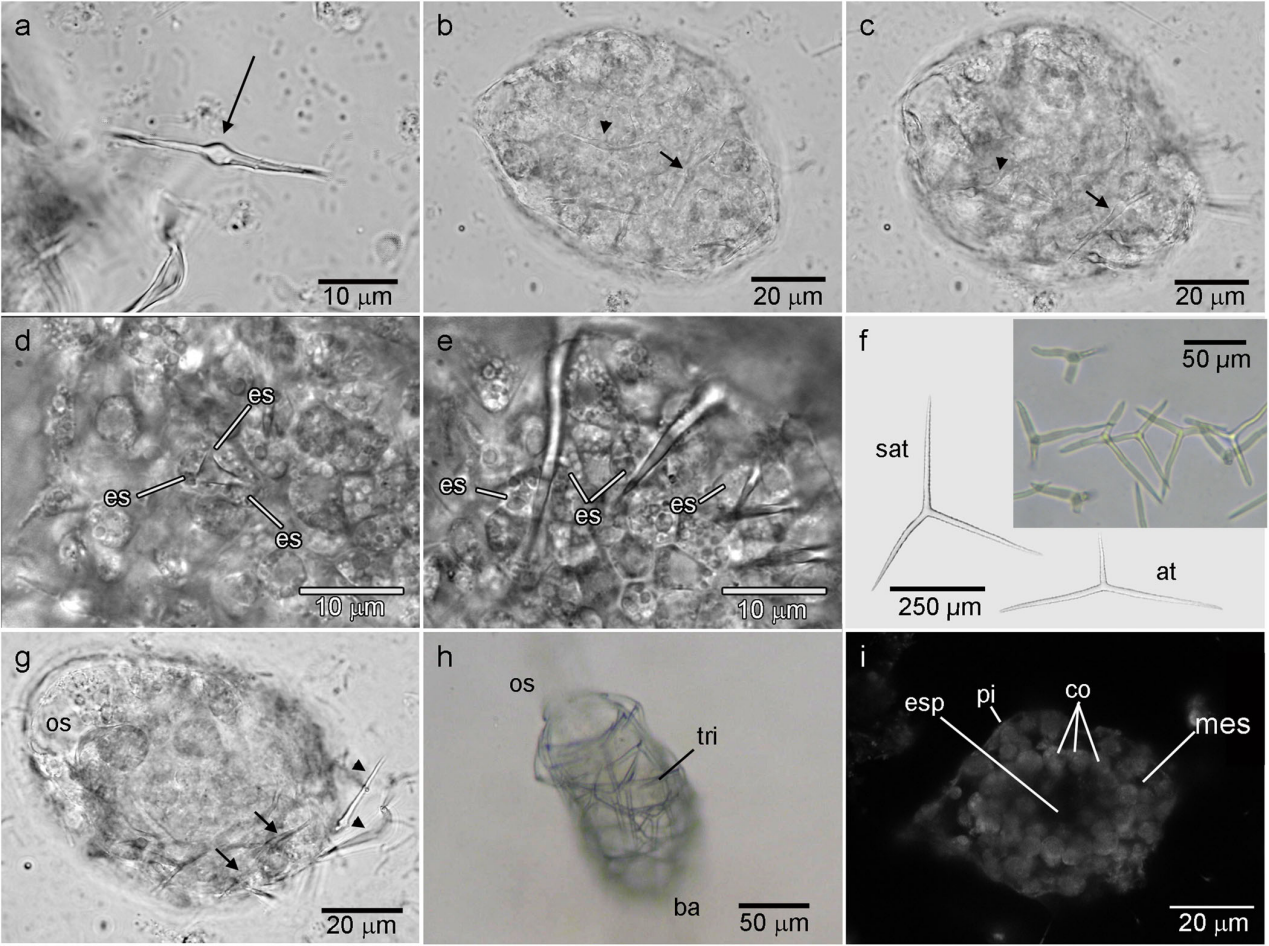
Formation of the juvenile skeleton of *Paraleucilla magna*. (a) Frontal view of a diactine with median‐swelling (differential interference contrast: DIC). (b) Diactines (arrow and arrowhead) formed in the inner cell mass concomitantly with the differentiation of the choanoderm (Phase contrast: PC). (c) The same diactines from ‘b’ being transported to the periphery of the juvenile (PC). (d) Beginning of the formation of triactines in the extracellular space of three sclerocytes (es) found in the inner cell mass (DIC). (e) Three to 4 h after the initiation of triactine formation, the spicule was transported to the edge of the juvenile (DIC). (f) Comparison of the different types of triactines found in the adult of *P. magna* (sat – subatrial skeleton triactine, at – atrial skeleton triactines) and in the olynthus (*insert*). (g) Expulsion of diactines from the juvenile (arrowhead) (DIC). (h) Olynthus approximately 7 days after settlement with a cortical skeleton formed by triactines (tri) and a well‐defined osculum (os) (ba – base) (PC). (i) Micrograph of a virtual tangential section made by confocal laser scanning microscope showing the spongocoel of the olynthus (co – choanocyte, esp – spongocoel, mes – mesohyl, pi – pinacocytes).

The first spicules were secreted concurrently with the formation of the primordial aquiferous system. The first type of spicule to be synthesized was diactines with a median‐swelling (Figure 4a). Several of these spicules were synthesized simultaneously by sclerocytes that differentiated in the ICM (Figure 4b). The diactines were initially immersed in the ICM but were subsequently transported to the periphery of the juvenile (Figure 4c).

During the formation of these first spicules, the juvenile had not yet begun to elongate its osculum‐base axis (Figure 4c). The triactines started to be synthesized between the 1st and 2nd days after settlement. The triactines were also initially synthesized in the ICM. This spiculogenesis began with three sclerocytes secreting a triangular crystalloid structure (Figure 4d and Supporting Information: Video S7), followed by the growth of the three actines. The sclerocytes that secreted the triactines also transported them to the peripheral region of the juvenile (Figure 4e and Supporting Information: Video S7). The triactines found in the juvenile were similar to tripods (with the center of the spicule elevated relative to the tips of the actines) and different from the triactines found in adults (Figure 4f). While the triactines were being produced, the diactines were restricted to the base of the sponge and were then expelled from the juvenile's body (Figure 4g). Interestingly, in some experiments, we left some juveniles to develop in Petri dishes with a small volume of water (~3 mL). We observed that some of them failed to secrete the diactines in the usual time (ca. 9 h), but after water renewal, they began secreting triactines instead of first forming diactines.

Approximately 5 days after settlement, the juveniles already exhibited a cortical skeleton and a well‐defined osculum‐base axis (apical‐basal) (Figure 4h), while maintaining an aquiferous system similar to the asconoid (Figures 3c and 4i). At this stage, the sack‐shaped choanoderm (covering the inner cavity of the entire sponge) was externally covered by pinacocytes (Figure 3c), and basopinacocytes secreting amorphous material that adhered the olynthus to the substrate became more evident (Figure 3d).

The juveniles maintained a cylindrical shape until approximately 16 days after settlement, when the base of the sponge widened and the first choanocyte chambers were formed by the apparent invagination of the choanoderm into the spongocoel (Figure 4e,e'). The invaginated region moved until it reached the opposite side of the sponge, isolating choanocytes in a spherical choanocyte chamber (Figure 3f). These choanocytes apparently divided, as the choanoderm grew previously to engage in another invagination to isolate another portion of choanocytes in a new choanocyte chamber (Figure 3g). The invaginations continued until a leuconoid aquiferous system, with rounded choanocyte chambers were formed (Figure 3h).

**Figure 5:**
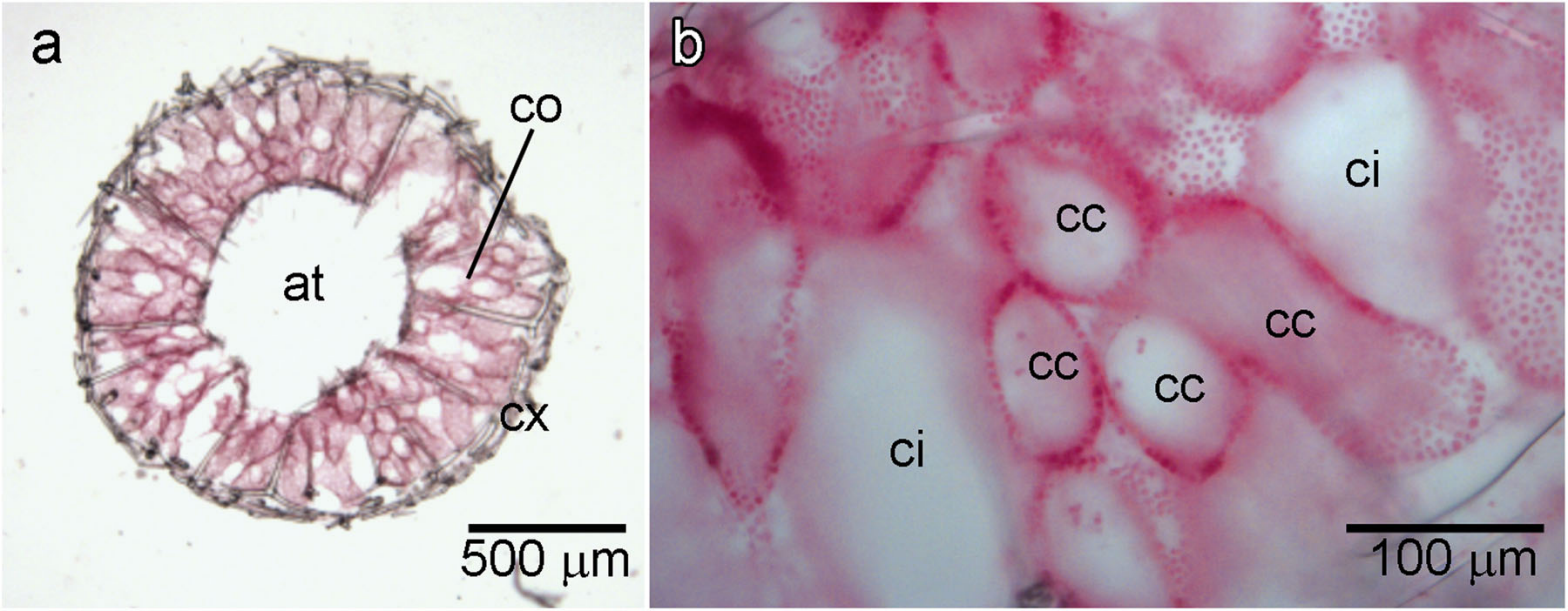
Leuconoid aquiferous system of a juvenile *Paraleucilla magna* approximately eight weeks old (cultivated in the field). (a) Cross‐section showing the cortical region (cx), choanosome (co), and atrium (at). (b) Detail of the choanosome (co) composed of inhalant canals (ci) and spherical to elongated choanocyte chambers (cc).

Later, we observed that the chambers formed by the invagination of the choanoderm were not directly in contact with each other. We found some cells (likely pinacocytes) covering the choanocyte chamber at the external portions of the choanocyte chambers. Therefore, we are interpreting that this morphogenesis led to the formation of the inhalant canals (Figure 3i). At this stage, the chambers opened into a spongocoel still lined with choanocytes, and water exits through the apical osculum. On the other hand, individuals collected on recruitment plates left in the field with a maximum of 8 weeks of life already presented a typical aquiferous system of the leuconoid type, with a wide atrium, well‐developed inhalant canals, and rounded choanocyte chambers (Figure 5). The formation of the atrium lined with pinacocytes could not be observed in vitro; however, it is possible that it occurs through the loss of choanocytes from the spongocoel during the sponge's growth.

## 4 Discussion

After the spawning, the post‐embryonic development of both calcareous sponges *S. hastifera* and *P. magna* started with an invagination of the anterior, ciliated pole into the cavity of the larva, and the attachment on the substrate with the spreading of the former granular cells of the posterior pole. The ciliated cells formed an inner cell mass that gave rise to sclerocytes, lophocytes, and choanocytes, while granular cells formed the pinacocytes. Independently of the aquiferous system type found in the adults of each species, the initial aquiferous system of the olynthus was asconoid, and the first spicules secreted were diactines. Later, some likely epithelial morphogenesis gave rise to the different heterocoelic aquiferous system of the species.

The release of larvae in both species did not seem to be linked to any specific period of the day, as they were released about 2–3 h after collection, regardless of the time when they were collected. The release in both species may be a result of the stress caused by removing the sponge from its natural environment. On two occasions, *S. hastifera* specimens released larvae only in the following morning after collection. This may be more related to an intrinsic characteristic of the individual (such as a greater resistance of the sponge to stressful conditions or the need for more time to complete embryogenesis) rather than any environmental influence. The difference in larval incubation methods may also have influenced their release. *Paraleucilla magna* incubates larvae in the epilarval trophocytes epithelium for a longer period (Lanna and Klautau 2012; Lanna et al. 2015), and under stressful conditions, the larvae, even if not fully mature, could be released. In *S. hastifera*, however, the larvae hatch immediately after inversion (Lanna and Klautau 2022), making it more difficult to collect animals with mature larvae ready to be released.

Centrifugation of larvae is an important step to concentrate them to carry out different types of experiments. Therefore, we empirically tested the effect of this procedure on the larvae of *S. hastifera* and *P. magna*. We observed that the centrifugation of larvae, in most cases, did not seem to affect the normal development of individuals. In some instances, at the end of centrifugation, the larvae did not exhibit normal morphology or typical swimming behavior, but after a short period, they regained both the shape and behavior characteristics of these larvae. In cases where normal conditions were not restored, the failure in development could be explained by other factors, such as nutrition or the developmental stage of the larva (immature or mature), rather than centrifugation (Jones 1979). On the other hand, in *Grantia compressa* (Fabricius, 1780), the centrifugation of larvae affected the shape, swimming, and behavior of most individuals concentrated by this method (Jorgensen 1918). Therefore, caution should be exercised when using centrifugation to obtain a larger number of larvae. We believe that each species may react differently to the effects of increased gravity.

### 4.1 Metamorphosis

The metamorphosis of *S. hastifera* and *P. magna* was not significantly different from what has already been observed in other species of Calcaronea (Amano and Hori 1993; Eerkes‐Medrano and Leys 2006; Ereskovsky 2010). Unlike the findings of Leys (2005), passing through “Haeckel's gastrula” stage was not a rare event during the metamorphosis of *S. hastifera* or *P. magna*. What we observed (and agree with the proposal previously made by those authors) is that this event is very quick (< 10 min) and occurs concomitantly with the settlement on the substrate. This finding reinforces the importance of using in vivo observation techniques to better understand the processes leading to the formation of the sponge body plan.

As observed in other Calcaronea (Duboscq and Tuzet 1937; Amano and Hori 1993; Leys 2005), the invagination of ciliated cells in the cavity of the amphiblastulae of *S. hastifera* and *P. magna* was apparently followed by dedifferentiation of these cells, followed by redifferentiation into choanocytes, sclerocytes, and ameboid cells within the post‐larva. At the same time, the cells derived from the granular cells remained covering the juvenile, dividing during sponge growth. Apparently, one could suggest that the cell fate of these sponges could be determined during the metamorphosis, but without a proper cell‐tracking experiment, such as those carried out by Nakanishi et al. (2014), we cannot state with certainty the cell fate of the granular and ciliated cells of the amphiblastulae of *S. hastifera* and *P. magna*.

### 4.2 Development of the Heterocoelic Aquiferous System

During the morphogenesis of the aquiferous system of *S. hastifera* (syconoid) and *P. magna* (leuconoid), both species pass through an initial organization similar to the asconoid (olynthus stage). The morphogenesis of the primordial choanoderm in both species occurs through a process of cavitation. Cavitation is a ubiquitous process in metazoan morphogenesis and can happen either through the rearrangement of cells or through programmed cell death at the center of the region where the cavity will be formed (Slack 2006). In the species investigated here, in other calcareous sponges, and even in some demosponges (e.g., *Halisarca dujardini* Johnston, 1892), this initial cavitation is more associated with cell rearrangement (mesenchymal‐epithelial transformation) than with cell death (Ereskovsky 2010). Mesenchymal‐epithelial transformations may be involved in the origin of animal multicellularity, considering that the epithelium is considered the basic unit of a metazoan (Leys and Riesgo 2012). However, as the molecular mechanisms regulating this event are still unknown for sponges, future studies focusing on this morphogenesis will help elucidate the evolution of this important morphogenetic process in metazoans.

**Figure 6:**
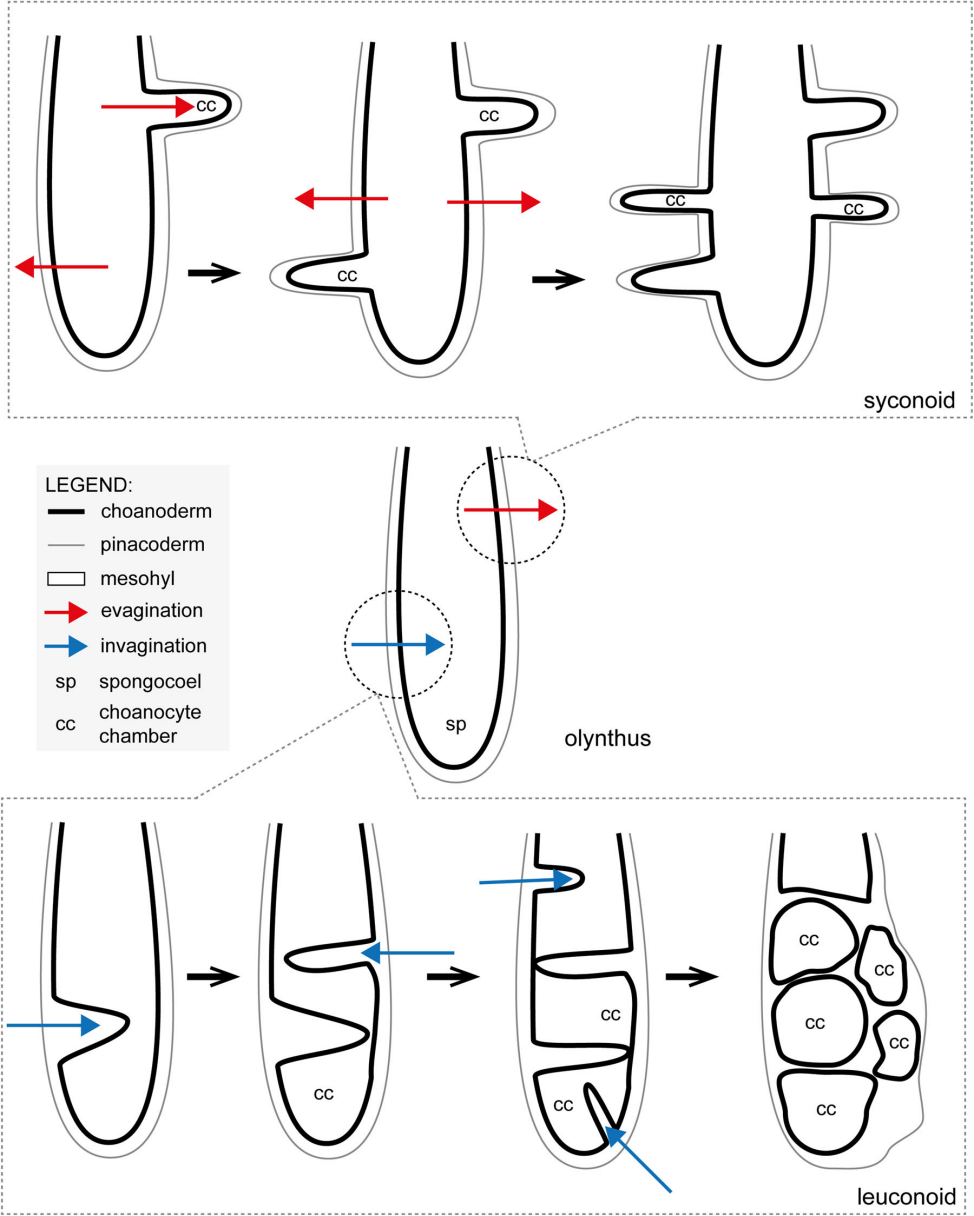
Diagram indicating the different vectors of the choanoderm folding to generate the heterocoelic aquiferous system in calcareous sponges. While in the leuconoid aquiferous the choanoderm folds inward the spongocoel (blue arrow), it folds outwards the spongocoel to form the syconoid aquiferous system (red arrow).

The formation of the heterocoelic aquiferous system in *P. magna* (and apparently in *S. hastifera*) seems to occur through epithelial morphogenesis. To form the choanocyte chambers of the juveniles, the choanoderm lining the primordial choanocyte chamber undergoes a series of invaginations (i.e., folding of the choanoderm occurs towards the spongocoel) in *P. magna*; while in *S. hastifera*, the choanoderm folds towards the external region of the sponge (evagination). Besides our current observations, there is only one study that described the formation of the heterocoelic aquiferous system in Calcarea, conducted at the end of the 19th century with *Sycon raphanus* Schmidt, 1862 (Maas 1900). In that species, the mechanism that leads to the formation of the syconoid choanocyte chambers is also the evagination of the choanoderm (Maas 1900). Leininger et al. (2014) did not describe the metamorphosis and development of the aquiferous system but state that the choanocyte chambers of the syconoid *Sycon ciliatum* (Fabricius, 1780) bud from the olynthus' choanoderm. Based on these observations, the formation of the heterocoelic aquiferous system in Calcaronea seems to be related to epithelial morphogenesis that will show different vectors depending on the organization being generated: outward from the spongocoel in the formation of syconoid systems and inward the spongocoel in leuconoid systems (Figure 6).

Invagination and evagination are morphogenetic processes also present at various stages of metazoan development. Epithelial morphogenesis is caused by changes in the shape of a few epithelial cells due to cytoskeletal rearrangements (a constriction in the cytoskeleton at the apical region of cells at the bending point of the epithelium will generate an invagination, while at the basal region it will cause an evagination) (Wang et al. 2022). Before forming the wedge‐shaped epithelium, cells tend to accumulate F‐actin and myosin II in the cytoplasm and to reinforce intercellular connections. Depending on where F‐actin is accumulated, the epithelium may fold inward or outward (Wang et al. 2022). It is important to note that, apart from the cytoskeleton dynamics modulated by the actomyosin system, the extracellular environment also play an important role. For instance, laminin and focal adhesion kinase (FAK) found in the extracellular matrix are important during the evagination (Wang et al. 2022). Porifera (except Homoscleromorpha) has been characterized by the absence of “true epithelia” as *zonula adherens* junctions are not common, and a *bona fide* basal membrane is absent (Tyler 2003). However, Leys and Riesgo (2012) challenged Tyler (2003) definition by showing that sponges possess functional and molecular characteristics that allow the classification of Porifera epithelia as “true”, at least in the broad sense of epithelium (Nickel et al. 2011). The results presented here and the ultrastructure of the adults, embryos, and larvae of these species (e.g., Lanna and Klautau 2012, 2022) support Leys and Riesgo's (2012) view, as, at least functionally, the choanoderm behaves as a proper epithelium during the morphogenesis of the choanocyte chambers. However, studies investigating the existence and spatiotemporal expression patterns of genes related to epithelial morphogenesis during the development of the heterocoelic aquiferous system in Calcarea are still needed to better understand this type of morphogenesis.

In *Sycon raphanus*, the formation of the apopyle occurs through the migration of cells located more basally in the choanoderm toward the region of the opening created by the evagination. This event occurs simultaneously with the evagination of the choanoderm that forms the choanocyte chamber. With the subsequent formation of new chambers, the cells that formed the apopyle divide and eventually line the internal cavity of the sponge, forming the atrium (Maas 1900). It was not possible to observe the formation of exhalant canals, apopyles, or even the atrium in the species studied here. Initially, the spherical chambers of *P. magna* would open directly into the region of the spongocoel, which had not yet undergone invagination (Figure 3g). However, based on the data reported by Maas (1900) and the results presented in Lanna and Klautau (2019), we think that the atrium lined by endopinacocytes will be formed through in situ transdifferentiation (metaplasia) of choanocytes into endopinacocytes in this region, but further investigations are necessary to confirm this hypothesis.

The formation of the leuconoid aquiferous system in *P. magna* could, in part, support one of the hypotheses for the diversification of the aquiferous system types in Calcarea presented by Dendy (1893). According to Dendy (1893), leuconoid sponges could derive from syconoid sponges that segmented the elongated chambers by the differentiation of choanocytes into pinacocytes. The formation of the leuconoid system in *P. magna*, although it does not “recapitulate” the syconoid aquiferous system, seems to occur through segmentation (through invaginations) of the continuous choanoderm of the olynthus. It is important to emphasize that Dendy (1893) did not believe that segmentation alone was responsible for generating the leuconoid aquiferous system. Indeed, he proposed that the leuconoid system found in various genera of Calcarea arose independently in different evolutionary lineages.

Phylogenies using molecular data have supported the hypothesis that the leuconoid aquiferous system emerged independently at least four times in the phylogeny of Calcaronea (as well as independently in Calcinea and other groups of Porifera). However, the ancestor of some leuconoid groups of Calcaronea could have had a syconoid aquiferous system (Manuel et al. 2003; Dohrmann et al. 2006; Voigt et al. 2012). Studies using morphological, molecular, and functional techniques are still needed to determine whether there is a direct ontogenetic relationship between these two types of aquiferous systems. However, as syconoid and leuconoid aquiferous systems share an asconoid stage during their development, and invagination/evagination shares the same molecular control, it is possible that these transitions are not that difficult to transpose. Nevertheless, it is important to note that there is no recapitulation in the development of the leuconoid aquiferous system, that is, *P. magna* at no point passes through the syconoid stage that may have been present in one of its ancestors.

A possible explanation for the “independent” emergence of the leuconoid aquiferous system along the phylogeny of Calcarea could be the misinterpretation (and subsequent misnaming) of the aquiferous systems found in this group, creating artifacts when interpreting phylogenetic trees (Cavalcanti and Klautau 2011). By aligning a better description of the morphology of the leuconoid system in these different lineages (as proposed by Cavalcanti and Klautau 2011) with the study of post‐embryonic development in these species, we may be able to confirm or refute the hypotheses suggesting that the aquiferous systems in Calcarea are homoplastic (e.g., Dohrmann et al. 2006; Manuel 2006; Voigt et al. 2012).

### 4.3 Spiculogenesis

While metamorphosis and the formation of the olynthus have been minimally investigated experimentally, various studies have already been conducted on the spiculogenesis of juvenile calcaroneans. The secretion of calcium carbonate spicules in *S. ciliatum* is dependent on the concentration of calcium ions in the water and is related to protein synthesis (Ledger and Jones 1977; Jones 1970, 1979). Moreover, different copies of α‐carbonic anhydrase genes are found in the genome of *S. ciliatum*, being expressed differentially in terms of quantity and timings for the development of the different spicule types found in the individuals (Voigt et al. 2014). We observed that juveniles of *P. magna* developing in Petri dishes with a small volume of water (~3 mL) failed to secrete their first spicules at the usual time (around 9 h), but after water renewal, they began secreting triactines instead of first forming diactines as usual. It is likely that the relative reduction of calcium ion availability affected their development. The reprogramming of spiculogenesis could introduce morphological variability in species, potentially leading to the emergence of “new species”. It was already demonstrated experimentally with the demosponge *Crambe crambe* (Schmidt, 1862) that different concentrations of silica can stimulate the sponges to produce different types of spicules (Maldonado et al. 1999). Based on the current findings, we hypothesize that changes in the concentration of calcium in the environment could select individuals that accelerate the production of triactines, suppressing the production of diactines, and creating new possibilities to form the primary skeleton of juveniles. In fact, this temporal shift occurs during the somatic embryogenesis (Lanna and Klautau 2019) and also after the metamorphosis of calciblastulae in Calcinea (Minchin 1900; Borojević 1969).

The establishment of model species in developmental biology studies is pragmatically related to the fact that the ontogeny of that species could serve as a model for other multicellular animals, but in practice, it also depends on the ease of reproduction, access to the species, and the specific questions researchers want to address (Tzika and Milinkovitch 2008). Due to characteristics such as symmetry, polarization, and several developmental events in Calcarea, these sponges are once again becoming important in research on the evolution of multicellular animals, as they were during the era of Haeckel (late 19th century) (Manuel 2006). In fact, the genome of *S. ciliatum* has proved that many genes related to the development of eumetazoans are present in representatives of this class (e.g., Leininger et al. 2014; Adamska 2018). The characterization of the post‐embryonic development of *S. hastifera* and *P. magna* in the present work will allow these species to be used as models in future experimental studies investigating gastrulation, epithelial formation, the development of the adult aquiferous system, or even sponge biomineralisation—events that were fundamental not only to the evolution of Porifera but also to the entire Metazoa.

## Author Contributions

**Emilio Lanna:** conceptualization, investigation, writing – original draft, methodology, validation, visualization, writing – review and editing, formal analysis, data curation. **Michelle Klautau:** supervision, resources, project administration, formal analysis, methodology, validation, visualization, writing – review and editing, writing – original draft, funding acquisition, investigation, conceptualization.

## Conflicts of Interest

The authors declare no conflicts of interest.

## Supporting information

Video S1. Real‐time videos of amphiblastula larvae of *S. hastifera* and *P. magna* swimming in Petri dishes.

Video S2. Real‐time video of the invagination of the ciliated (anterior) pole of the amphiblastula of *P. magna* moments before starting to attach to the substrate. This moment was coined as “Haeckel's gastrula”.

Video S3. Time‐lapse video with details of the granular cells of the amphiblastula of *P. magna* attaching and spreading on the substrate. The clock in the upper left corner denotes the elapsed time spreading on the surface.

Video S4. Time‐lapse video with details of the movement in the inner cell mass and the differentiation of the pinacocytes and sclerocytes in the post‐metamorphic larva of *P. magna*. First spicules being secreted (diactines) and the subsuperficial space appearance in the post‐larva. The clock in the upper right corner denotes the elapsed time.

Video S5. Real‐time video with details of the newly differentiated choanocytes in the olynthus of *P. magna* showing the flagella beating without synchrony and uniform direction. The clock in the upper left corner denotes the elapsed time.

Video S6. Time‐lapse video showing the differentiation of the inner cell mass in choanoderm of the olynthus of *P. magna*. The clock in the upper left corner denotes the elapsed time.

Video S7. Time‐lapse video showing the secretion and transport of triactines in the post‐larva. Sclerocytes were the cells responsible for both secretion, elongation, and transportation of the spicule in the juvenile of *P. magna*.

## Data Availability

The data that support the findings of this study are available from the corresponding author upon reasonable request.

## References

[mrd70060-bib-0001] Adamska, M. 2018. “Differentiation and Transdifferentiation of Sponge Cells.” In Marine Organisms as Model Systems in Biology and Medicine, edited by M. Kloc and J. Kubiak , 229–253. Springer International Publishing. 10.1007/978-3-319-92486-1_12 30083923

[mrd70060-bib-0002] Amano, S. , and I. Hori . 1993. “Metamorphosis of Calcareous Sponges 2. Cell Rearrangement and Differentiation in Metamorphosis.” Invertebrate Reproduction & Development 24, no. 1: 13–26.

[mrd70060-bib-0004] Bidder, G. P. 1898. “The Skeleton and Classification of Calcareous Sponges.” Proceedings of the Royal Society of London B 64: 61–76.

[mrd70060-bib-0005] Borojević, R. 1969. “Étude Du Développement Et De La Différenciation Cellulaire D'éponges Calcaires Calcinéennes (Genres *Clathrina* et *Ascandra*).” Annales d'Embryologie et de Morphogenèse 2, no. 1: 15–36.

[mrd70060-bib-0006] Borojević, R. , N. Boury‐Esnault , M. Manuel , et al. 2002. “Order Leucosolenida Hartman, 1958.” In Systema Porifera: A Guide to the Classification of Sponges, edited by J. N. A. Hooper , R. W. M. van Soest , 2, 1157–1184. Kluwer Academic/Plenum Publishers.

[mrd70060-bib-0007] Cavalcanti, F. F. , and M. Klautau . 2011. “Solenoid: A New Aquiferous System to Porifera.” Zoomorphology 130, no. 4: 255–260. 10.1007/s00435-011-0139-7.

[mrd70060-bib-0008] Dohrmann, M. , O. Voigt , D. Erpenbeck , and G. Wörheide . 2006. “Non‐Monophyly of Most Supraspecific Taxa of Calcareous Sponges (Porifera, Calcarea) Revealed by Increased Taxon Sampling and Partitioned Bayesian Analysis of Ribosomal DNA.” Molecular Phylogenetics and Evolution 40: 830–843. 10.1016/j.ympev.2006.04.016. 16762568

[mrd70060-bib-0009] Duboscq, O. , and O. Tuzet . 1937. “L'ovogenèse, La Fécondation Et Les Premiers Stades Du Développement Des Éponges Calcaires.” Archives de Zoologie Expérimentale et Générale 79: 157–316.

[mrd70060-bib-0010] Eerkes‐Medrano, D. I. , and S. P. Leys . 2006. “Ultrastructure and Embryonic Development of a Syconoid Calcareous Sponge.” Invertebrate Biology 125, no. 3: 177–194. 10.1111/j.1744-7410.2006.00051.x.

[mrd70060-bib-0011] Ereskovsky, A. V. 2010. The Comparative Embryology of Sponges. Springer.

[mrd70060-bib-0012] Ereskovsky, A. V. , D. B. Tokina , C. Bézac , and N. Boury‐Esnault . 2007. “Metamorphosis of Cinctoblastula Larvae (Homoscleromorpha, Porifera).” Journal of Morphology 268: 518–528. 10.1002/jmor.10506. 17427974

[mrd70060-bib-0013] Gilbert, S. , and M. J. F. Barresi . 2016. Developmental Biology 11e. Sinauer Associates.

[mrd70060-bib-0014] Gonobobleva, E. , and A. V. Ereskovsky . 2004. “Metamorphosis of the Larva of *Halisarca dujardini* (Demospongiae, Halisarcida).” Bulletin de L'Institut Royal des Sciences Naturelles de Belgique, Biologie 74: 101–115. (impresso).

[mrd70060-bib-0015] Haeckel, E. 1872. Die Kalkschwämme, Eine Monographie (1–3). Verlag von Georg Reimer.

[mrd70060-bib-0016] Hall, B. K. , and M. H. Wake . 1999. The Origin and Evolution of Larval Forms. Academic Press.

[mrd70060-bib-0017] Jones, W. C. 1970. “The Composition, Development, Form and Orientation of Calcareous Sponge Spicules.” In The Biology of the Porifera, edited by W. G. Fry , 91–123. Academic Press.

[mrd70060-bib-0018] Jones, W. C. 1979. “Spicule Growth and Production in Juvenile *Sycon ciliatum* .” In Colloques Internationaux du CNRS, Biologie des Spongiaires, edited by C. Levi and N. Boury‐Esnault , 67–77. CNRS.

[mrd70060-bib-0019] Jorgensen, O. M. 1918. “Note on the Larva of *Grantia compressa* .” Report of the Dove Marine Laboratory 7: 60–61.

[mrd70060-bib-0020] Lanna, E. , and M. Klautau . 2010. “Oogenesis and Spermatogenesis In *Paraleucilla magna* (Porifera, Calcarea).” Zoomorphology 129, no. 4: 249–261. 10.1007/s00435-010-0117-5.

[mrd70060-bib-0021] Lanna, E. , and M. Klautau . 2012. “Embryogenesis and Larval Ultrastructure in *Paraleucilla magna* (Calcarea, Calcaronea), With Remarks on the Epilarval Trophocyte Epithelium (‘Placental Membrane’).” Zoomorphology 131, no. 4: 277–292. 10.1007/s00435-012-0160-5.

[mrd70060-bib-0022] Lanna, E. , and M. Klautau . 2018. “Life History and Reproductive Dynamics of the Cryptogenic Calcareous Sponge *Sycettusa hastifera* (Porifera, Calcarea) Living in Tropical Rocky Shores.” Journal of the Marine Biological Association of the United Kingdom 98, no. 3: 505–514. 10.1017/S0025315416001466.

[mrd70060-bib-0023] Lanna, E. , and M. Klautau . 2019. “The Choanoderm of *Sycettusa hastifera* (Calcarea, Porifera) Is Able to Generate New Individuals.” Invertebrate Biology 138, no. 3: e12262. 10.1111/ivb.12262.

[mrd70060-bib-0024] Lanna, E. , and M. Klautau . 2022. “Oogenesis and Embryogenesis in a Cryptogenic Species of Calcareous Sponge (Calcaronea, Heteropiidae) in the Southwestern Atlantic.” Invertebrate Biology 141, no. 2: e12375. 10.1111/ivb.12375.

[mrd70060-bib-0025] Lanna, E. , R. Paranhos , P. C. Paiva , and M. Klautau . 2015. “Environmental Effects on the Reproduction and Fecundity of the Introduced Calcareous Sponge *Paraleucilla magna* in Rio de Janeiro, Brazil.” Marine Ecology 36: 1075–1087. 10.1111/maec.12202.

[mrd70060-bib-0026] Ledger, P. , and W. C. Jones . 1977. “Spicule Formation in the Calcareous Sponge *Sycon ciliatum* .” Cell and Tissue Research 181, no. 4: 553–567. 884721 10.1007/BF00221776

[mrd70060-bib-0027] Leininger, S. , M. Adamski , B. Bergum , et al. 2014. “Developmental Gene Expression Provides Clues to Relationships Between Sponge and Eumetazoan Body Plans.” Nature Communications 5: 3905. 10.1038/ncomms4905. 24844197

[mrd70060-bib-0028] Leys, S. P. 2005. “Gastrulation in Calcareous Sponges: In Search of Haeckel's Gastraea.” Integrative and Comparative Biology 45: 342–351. 10.1093/icb/45.2.342. 21676779

[mrd70060-bib-0029] Leys, S. P. , L. Grombacher , D. Field , et al. 2025. “A Morphological Cell Atlas of the Freshwater Sponge *Ephydatia muelleri* With Key Insights From Targeted Single‐Cell Transcriptomes.” Evodevo 16, no. 1: 1. 39953556 10.1186/s13227-025-00237-7 PMC11827373

[mrd70060-bib-0030] Leys, S. P. , and A. Riesgo . 2012. “Epithelia, An Evolutionary Novelty of Metazoans.” Journal of Experimental Zoology Part B: Molecular and Developmental Evolution 318B, no. 6: 438–447. 10.1002/jez.b.21442. 22057924

[mrd70060-bib-0031] Lopes, M. V. , and M. Klautau . 2023. “Phylogeny and Revision of *Leucaltis* and *Leucettusa* (Porifera: Calcarea), With New Classification Proposals and Description of a New Type of Aquiferous System.” Zoological Journal of the Linnean Society 198, no. 3: 691–746. 10.1093/zoolinnean/zlad008.

[mrd70060-bib-0032] Maas, O. 1900. “Die Weiterentwicklung der Syconen Nach Der Metamorphose.” Zeitschrift für Wissenschaften Zoologische 67: 215–240.

[mrd70060-bib-0033] Maldonado, M. , M. C. Carmona , M. J. Uriz , and A. Cruzado . 1999. “Decline in Mesozoic Reef‐Building Sponges Explained by Silicon Limitation.” Nature 401: 785–788. 10.1038/44560.

[mrd70060-bib-0034] Manuel, M. 2006. “Phylogeny and Evolution of Calcareous Sponges.” Canadian Journal of Zoology 84: 225–241. 10.1139/z06-005.

[mrd70060-bib-0035] Manuel, M. , C. Borchiellini , E. Alivon , Y. Le Parco , J. Vacelet , and N. Boury‐Esnault . 2003. “Phylogeny and Evolution of Calcareous Sponges: Monophyly of Calcinea and Calcaronea, High Level of Morphological Homoplasy, and the Primitive Nature of Axial Symmetry.” Systematic Biology 52, no. 3: 311–333. 12775522 10.1080/10635150390196966

[mrd70060-bib-0036] Minchin, E. A. 1900. “Chapter III. Sponges ‐ Phylum Porifera.” In A Treatise on Zoology, edited by R. Lankester , 1–178. Adam & Charles Black.

[mrd70060-bib-0037] Nakanishi, N. , S. Sogabe , and B. M. Degnan . 2014. “Evolutionary Origin of Gastrulation: Insights From Sponge Development.” BMC Biology 12, no. 1: 26. 10.1186/1741-7007-12-26. 24678663 PMC4021757

[mrd70060-bib-0038] Nickel, M. , C. Scheer , J. U. Hammel , J. Herzen , and F. Beckmann . 2011. “The Contractile Sponge Epithelium Sensu Lato ‐ Body Contraction of the Demosponge *Tethya wilhelma* Is Mediated by the Pinacoderm.” Journal of Experimental Biology 214, no. 10: 1692–1698. 10.1242/jeb.049148. 21525315

[mrd70060-bib-0041] Simpson, T. L. 1984. The Cell Biology of Sponges. Springer‐Verlag.

[mrd70060-bib-0042] Slack, J. M. W. 2006. Essential Developmental Biology, 365. Blackwell Publishing.

[mrd70060-bib-0043] Tuzet, O. 1973. “Éponges Calcaires.” In Traité de Zoologie ‐ Spongiaires ‐ Anatomie, Physiologie, Systématique, Écologie, edited by P.‐P. Grassé , III, 27–132. Masson et Cie.

[mrd70060-bib-0044] Tyler, S. 2003. “Epithelium ‐The Primary Building Block for Metazoan Complexity.” Integrative and Comparative Biology 43, no. 1: 55–63. 10.1093/icb/43.1.55. 21680409

[mrd70060-bib-0045] Tzika, A. C. , and M. C. Milinkovitch . 2008. “A Pragmatic Approach for Selecting Evo‐Devo Model Species in Amniotes.” In Evolving Pathways: Key Themes in Evolutionary Developmental Biology, edited by A. Minelli and G. Fusco , 123–143. Cambridge University Press.

[mrd70060-bib-0046] Voigt, O. , M. Adamski , K. Sluzek , and M. Adamska . 2014. “Calcareous Sponge Genomes Reveal Complex Evolution of α‐carbonic Anhydrases and Two Key Biomineralization Enzymes.” BMC Evolutionary Biology 14, no. 1: 230. 10.1186/s12862-014-0230-z. 25421146 PMC4265532

[mrd70060-bib-0047] Voigt, O. , E. Wülfing , and G. Wörheide . 2012. “Molecular Phylogenetic Evaluation of Classification and Scenarios of Character Evolution in Calcareous Sponges (Porifera, Class Calcarea).” PLoS One 7, no. 3: e33417. 10.1371/journal.pone.0033417. 22479395 PMC3314023

[mrd70060-bib-0048] Wang, Y. , D. Stonehouse‐Smith , M. T. Cobourne , J. B. A. Green , and M. Seppala . 2022. “Cellular Mechanisms of Reverse Epithelial Curvature in Tissue Morphogenesis.” Frontiers in Cell and Developmental Biology 10: 1066399. 10.3389/fcell.2022.1066399. 36518538 PMC9742543

